# Trans-Species Polymorphism and Selection in the MHC Class II *DRA* Genes of Domestic Sheep

**DOI:** 10.1371/journal.pone.0011402

**Published:** 2010-06-30

**Authors:** Keith T. Ballingall, Mara S. Rocchi, Declan J. McKeever, Frank Wright

**Affiliations:** 1 Division of Epidemiology and Population Biology, Moredun Research Institute, Penicuik, United Kingdom; 2 Department of Pathology and Infectious Diseases, Royal Veterinary College, Hatfield, United Kingdom; 3 Biomathematics and Statistics, Scotland (BIOSS), Edinburgh, United Kingdom; Université de Toulouse, France

## Abstract

Highly polymorphic genes with central roles in lymphocyte mediated immune surveillance are grouped together in the major histocompatibility complex (MHC) in higher vertebrates. Generally, across vertebrate species the class II MHC *DRA* gene is highly conserved with only limited allelic variation. Here however, we provide evidence of trans-species polymorphism at the *DRA* locus in domestic sheep (*Ovis aries*). We describe variation at the *Ovar-DRA* locus that is far in excess of anything described in other vertebrate species. The divergent *DRA* allele (Ovar-DRA*0201) differs from the sheep reference sequences by 20 nucleotides, 12 of which appear non-synonymous. Furthermore, DRA*0201 is paired with an equally divergent *DRB1* allele (Ovar-DRB1*0901), which is consistent with an independent evolutionary history for the *DR* sub-region within this MHC haplotype. No recombination was observed between the divergent *DRA* and *B* genes in a range of breeds and typical levels of MHC class II DR protein expression were detected at the surface of leukocyte populations obtained from animals homozygous for the DRA*0201, DRB1*0901 haplotype. Bayesian phylogenetic analysis groups Ovar-DRA*0201 with *DRA* sequences derived from species within the Oryx and Alcelaphus genera rather than clustering with other ovine and caprine *DRA* alleles. Tests for Darwinian selection identified 10 positively selected sites on the branch leading to Ovar-DRA*0201, three of which are predicted to be associated with the binding of peptide antigen. As the Ovis, Oryx and Alcelaphus genera have not shared a common ancestor for over 30 million years, the DRA*0201 and DRB1*0901 allelic pair is likely to be of ancient origin and present in the founding population from which all contemporary domestic sheep breeds are derived. The conservation of the integrity of this unusual *DR* allelic pair suggests some selective advantage which is likely to be associated with the presentation of pathogen antigen to T-cells and the induction of protective immunity.

## Introduction

Components of the adaptive arm of the immune system emerged around the appearance of jawed vertebrates some 450 million years ago. Associated with these pivotal events in vertebrate evolution, multiple polymorphic loci with a variety of immunological functions appeared closely linked within the vertebrate genome [1]. Termed the major histocompatibility complex (MHC), this region encodes a range of MHC class I and class II cell surface glycoproteins with central roles in T cell mediated immune surveillance. MHC class I and II molecules present pathogen-derived peptide fragments for recognition by antigen specific T cells, resulting in their clonal expansion and differentiation into effector and memory cells [2].

The MHC includes the most polymorphic protein-encoding loci in vertebrates with allelic diversity linked to codons encoding amino acids associated with the binding of peptide antigen. The substantial allelic diversity observed at MHC loci within populations is thought to be maintained by some form of balancing selection (heterozygous advantage and or frequency dependent selection) arising from the requirement to recognise and respond to pathogens that constantly evolve to evade the hosts immune response [3], [4]. A number of mechanisms have been suggested to be responsible for generating diversity at MHC loci, including accumulation of point mutations coupled with recombination between alleles and loci [5]. Here we provide evidence that ancient trans-species allelic lineages have contributed towards unusual allelic diversity within the MHC of domestic sheep.

The MHC of sheep (OLA) and cattle (BoLA) share orthologous class II *DR* and *DQ A* and *B* loci with rodents and primates. A single dominant and highly polymorphic *DRB* locus encoding the beta chain of the MHC class II DR heterodimer has been described in domestic sheep (*Ovis aries*, *Ovar-DRB1*), [6], [7] and cattle (*BoLA-DRB3*, [8], [9]. The *DRA* locus, which encodes the alpha chain of the DR heterodimer is closely linked to *DRB* and considered almost monomorphic: it is therefore rarely targeted for comprehensive analysis. However, two *DRA* allele sequences have been described in domestic sheep [10], [11] that differ by five substitutions within the coding region, four of which are non-synonymous. In contrast, no allelic diversity is associated with three independently isolated *Bos taurus* cattle *DRA* cDNA and genomic clones [12]–[14] and only 3 minor alleles, each with a single synonymous substitution have been described in a recent analysis of *DRA* exon 2 diversity in 384 *B. taurus* dairy cattle [15].

**Figure 1:**
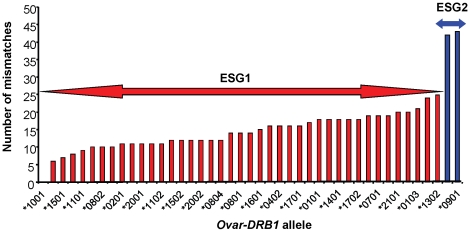
Diversity of *Ovar-DRB1* alleles associated with ESG1 and ESG2. Nucleotide mismatch analysis of 42 *Ovar-DRB1* alleles held in the sheep immunopolymorphism (IPD) data base www.ebi.ac.uk/ipd/mhc/ovar/index.html showing the degree of diversity between alleles represented in ESG1 and ESG2.

We have recently detailed unusual patterns of diversity at coding, intronic and regulatory regions of *Ovar-DRB1* alleles [7] representing each of two evolutionary separated allelic groups, (ESG) [16]. The extent of diversity between ESGs is consistent with allelic lineages with independent evolutionary histories that may be the result of ancient cross-species hybridisation [16]. ESG 1 includes all but 2 of the 60 *Ovar-DRB1* alleles present within the sheep IPD-MHC data base, http://www.ebi.ac.uk/ipd/mhc/ovar/index.html, while ESG 2 includes the remaining two alleles within the Ovar-DRB1*09 allelic family. The extent of sequence divergence between *DRB1* alleles representative of ESG1 and ESG2 is demonstrated in Figure 1.

Cross-species hybridisation events have been used to explain unusual features of the nuclear and mitochondrial genomes of a number of domestic animal species, including goats [17], cattle [18], [19], sheep [20] and chickens [21]. Such events leave characteristic footprints in the genome, such as phylogenetic incongruence between mitochondrial and nuclear genes, regions with high levels of single nucleotide polymorphism (SNPs) and multiple mitochondrial lineages. The leg colour of domestic chickens has been linked to such an event, with yellow legs derived not from the red jungle fowl (*Gallus gallus*) but from the grey jungle fowl (*Gallus sonneratii*), [21]. This is suggestive of a hybridisation event between the two species, with the genomic background of the grey having been diluted out through repeated crossing with red jungle fowl. Only fragments of the original grey jungle fowl genome remain, possibly due to human selection for particular phenotypic traits such as leg colour or through linkage to genes involved in resistance to disease.

Similarly, evidence has been provided that wild goats are hybrids between two ancestral species, one providing the nuclear genome and the other responsible for mitochondria adapted to high altitude [17].

Using sheep homozygous at the MHC and representative of both ESG1 and ESG2, we have extended this analysis to the closely linked and generally conserved *DRA* locus. The extent of the *DRA* diversity identified suggests that the DR sub region associated with ESG2 has evolved independently from ESG1 and is more likely to be an ancient allelic lineages preserved by balancing selection rather than evidence for a cross-species hybridisation event.

## Results

### Comparison of *DRA* diversity between sheep and cattle

**Figure 2:**
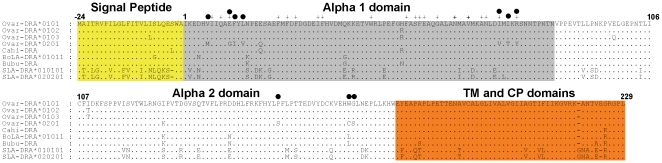
Multiple alignments of the predicted amino acid sequences. Alignment of sequences derived from four sheep *DRA* transcripts in comparison with the orthologous goat, cattle buffalo and pig sequences shown in Supplementary Figure S1. Sequences are numbered from the first amino acid of the mature protein. Amino acid identity is represented by dots (.) and amino acids predicted [47] to be associated with the peptide binding domain are highlighted with the + symbol. Ten sites under positive selection are labelled •. TM, transmembrane and CP, cytoplasmic domains.

Nucleotide and predicted amino acid sequences of full length *DRA* transcripts from eight sheep revealed significant allelic diversity (Supplementary Figure S1 and Figure 2). Four *DRA* alleles were identified and grouped into two allelic families, DRA*01 and DRA*02, based on the nomenclature described in the materials and methods. Comparison with the Ovar-DRA*0101 reference sequence revealed 3 substitutions associated with Ovar-DRA*0102, 5 associated with*0103, and 20 associated with the *0201 sequence. DRA*0201 was isolated from an animal homozygous for an MHC haplotype which includes DRB1*0901, an allele representative of ESG 2 (Figure 1), confirming that both *DRA* and *DRB* genes on this MHC haplotype have maintained patterns of diversity consistent with an independent evolutionary history.

In contrast, nucleotide and predicted amino acid sequences of full length *DRA* transcripts from six African and Asian *B. indicus* cattle revealed only two synonymous substitutions at positions 195 and 276 (Figure 2, Supplementary Figure S1 and summarised in Table 1), corresponding to the BoLA-DRA*01013 and *01014 alleles [20].

**Table 1:** Cattle *DRA/DRB3* genotypes.

Cattle			
Animal	Breed	*BoLA-DRA* allele	*BoLA-DRB3* PCR-RFLP
8340	Sahiwal	BoLA-DRA*01013 BoLA-DRA*01011	*31/37*
8375	Sahiwal	BoLA-DRA*01011	*06/47*
377	Boran	BoLA-DRA*01014	*32/34*
411	Boran	BoLA-DRA*01011	*27/15*
414	Boran	BoLA-DRA*01011	*11/22*
Br37	Boran	BoLA-DRA*01014	*31/23*

#### Recombination between DRA*0201 and DRB1*0901

To consolidate evidence for linkage between Ovar-DRA*0201 and DRB1*0901, genomic DNA from 56 animals representing a range of different breeds, including Scottish Blackface, Suffolk/Texel, Texel/British milk and Suffolk/British milk crosses, was screened for the DRB1*0901 allele. Six *0901 positive animals were identified across the different breeds, all of which were positive for DRA*0201 (Table 2). Ovar-DRA*0201 was not recorded in any of the remaining *0901 negative animals indicating that in this sample of MHC heterozygous sheep there was no evidence of recombination between DRA*0201 and DRB1*0901.

**Table 2:** Sheep *DRA/DRB1* haplotypes and genotypes.

Sheep				
Animal	Breed	*Ovar-DRA* allele	*Ovar-DRB1* allele	*DRB1* ESG
B284+	SB	Ovar-DRA*0101	Ovar-DRB1*0301	1
B414+	SB	Ovar-DRA*0201	Ovar-DRB1*0901	2
B564+	SB	Ovar-DRA*0101	Ovar-DRB1*0101	1
B209+	SB	Ovar-DRA*0102	Ovar-DRB1*0501	1
4040	BFL	Ovar-DRA*0102	Ovar-DRB1*0201 Ovar-DRB1*0601	1
4080	BFL	Ovar-DRA*0102	Ovar-DRB1*0801 Ovar-DRB1*0201	1
JD186*	SB	Ovar-DRA*0201 Ovar-DRA*0101	Ovar-DRB1*0901 Ovar-DRB1*0301	1 and 2
OPA11*	S/BM	Ovar-DRA*0201 Ovar-DRA*0102/3 #	Ovar-DRB1*0901 Ovar-DRB1*0102	1 and 2
OPA132*	BM/Tex	Ovar-DRA*0201 Ovar-DRA*0102/3	Ovar-DRB1*0901 Ovar-DRB1*1501	1 and 2
OPA184*	S/Tex	Ovar-DRA*0201 Ovar-DRA*0102/3	Ovar-DRB1*0901 Ovar-DRB1*0102	1 and 2
OPA198*	S/Tex	Ovar-DRA*0201 Ovar-DRA*0102/3	Ovar-DRB1*0901 Ovar-DRB1*0102	1 and 2
OPA200*	S/Tex	Ovar-DRA*0201 Ovar-DRA*0102/3	Ovar-DRB1*0901 Ovar-DRB1*0102	1 and 2
OPA219*	S/Tex	Ovar-DRA*0201 Ovar-DRA*0102/3	Ovar-DRB1*0901 Ovar-DRB1*1001	1 and 2

*DRA exon 2 is amplified from genomic DNA as described by Sena et al [36].

+ represents animals homozygous at the MHC.

# indicates that the sequence of exon 2 is unable to distinguish between alleles *0102 and *0103. ND, not determined. Sheep breeds and cross breeds are represented as follows; SB, Scottish Blackface; BFL, Blue faced Leicester; FL, Finnish landrace; AM, Australian Merino; S, Suffolk; BM, British Milk; Tex, Texel.

#### The Ovar-DRα protein

Of the 20 substitutions associated with the *0201 sequence, 12 were identified as non-synonymous (Figure 2). This level of diversity can be put in perspective by comparison with orthologous caprine and bovine DRα chains, which differ from the ovine reference sequence by only 5 and 9 amino acids respectively (Figure 2). This is despite estimates of twenty million years since domestic sheep and cattle shared a common ancestor.

A more comprehensive analysis of the distribution of diversity within the DRα*0201 molecule revealed that 8 of the 12 amino acid substitutions locate to the α1 domain while the remaining are found in the α2 domain (Figure 2). Of the 8 α1 domain substitutions, 3 are predicted to locate to the peptide binding site (PBS, Figure 2). This contrasts with all other sequences shown in Figure 2, where changes in amino acid sequence are predicted to fall outside the PBS. The functional significance of this diversity was investigated using SIFT BLink software which predicts the impact of each of the amino acid substitutions associated with Ovar-DRA*0201 when compared to the reference sequence Ovar-DRA*0101. The results for each substitution are shown in Table 3. The software predicts that the R76Y substitution located within the PBS, is likely to have significant impact on the protein structure and consequently the range of peptides presented to CD4^+^ T cells. Other substitutions which are predicted to impact protein function, such as V6M, appear adjacent to an amino acid predicted to be located within the PBS while W168C and G169S are located within the alpha 2 domain.

**Table 3:** Sorting intolerant from tolerant substitutions.

Substitution*	SIFT BLink Prediction	Score
V6M	Predicted to affect function	0.01*
E11G	Tolerated	0.09
F12I	Tolerated	0.19
L14V	Tolerated	0.20
E18Q	Tolerated	0.65
I72V	Tolerated	0.17
I74T	Tolerated	0.29
R76Y	Predicted to affect function	0.00*
I126K	Tolerated	1.00
P152S	Tolerated	0.67
W168C	Predicted to affect function	0.00*
G169S	Predicted to affect function	0.00*

*Amino acid substitutions are defined as follows; V6M, DRA*0101 amino acid V, position as shown in Figure 2, DRA*0201 substitution M. The score ranges between 0 and 1 where 0 is predicted to affect function and 1 is tolerated. A threshold cut off score of 0.05 is used to define the boundary between affect and tolerance.

### Phylogenetic analysis

**Figure 3:**
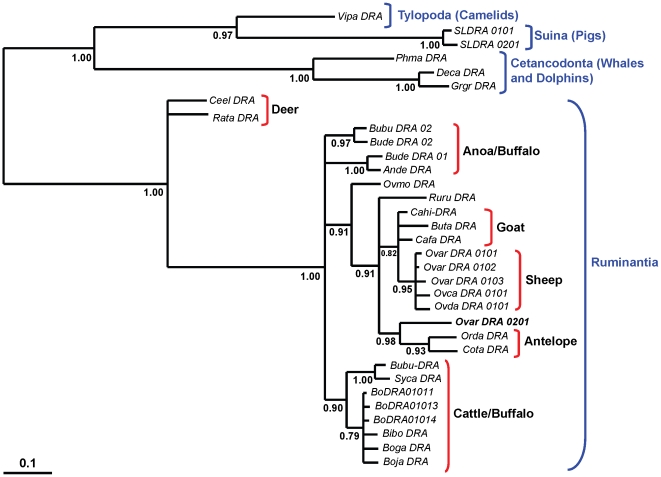
Bayesian phylogenetic tree estimating the relationships between 33 *Cetartiodactylia DRA* sequences. Species designations associated with the MHC nomenclature are as follows; Bibo, *Bison bonasus* (European Bison); Boga, *Bos gaurus* (Gaur); Boja, *Bos javanicus* (Banteng); Bubu, *Bubalus bubalis* (Asian Water Buffalo); Bude, *Bubalus depressicornis* (Lowland Anoa); Buta, *Budorcas taxicolor* (Takin); Cahi, *Capra hircus*, (Domestic Goat); Cafa, *Capra falconeri* (Markhor); Ceel, *Cervus elaphus* (Red Deer); Cota, *Connochaetes taurinus* (Blue Wildebeest); Deca, *Delphinus capensis* (Common Dolphin); Grgr, *Grampus griseus* (Risso's Dolphin); Orda, *Oryx dammah* (Scimitar Horned Oryx); Ovar, *Ovis aries* (Domestic Sheep); Ovca, *Ovis canadensis*, (Canadian Bighorn Sheep); Ovda, *Ovis dalli* (Dalli Sheep); Ovmo, *Ovibos moschatus* (Musk Ox); Phca, *Physeter catodon* (Sperm Whale); Ruru, *Rupicapra rupicapra* (Chamois); Rata, *Rangifer tarandus* (Reindeer); SLA, Swine Leucocyte Antigen (Pig); Syca, *Synserus caffer* (African Buffalo); Vipa, *Vicugna pacos* (Alpaca).

The origin of Ovar-DRA*0201 allele was analysed in greater detail using 33 *DRA* exon 2 sequences derived from 25 species within the order *Cetartiodactyla*. The four *Cetartiodactyla* sub orders; *tylopoda* (camelids), *suiformes* (pigs), *cetancodonta* (whales and dolphins) and *ruminanta* are each represented. A pair-wise alignment of these *DRA* sequences is shown in supplementary Figure S2. Bayesian phylogenetic trees were estimated using an alignment of the 11 full length *DRA* transcripts shown in Supplementary Figure S1 combined with the 33 *DRA* exon 2 sequences shown in Supplementary Figure S2. The tree topology obtained using only the 11 full length sequences is maintained in the combined analysis. The tree shown in Figure 3 estimates that Ovar-DRA*0201 diverged from the Ovar-DRA*01 family of alleles prior to the *Capra/Ovis* split, which is estimated to have occurred between 5 and 7 MYA, while the remaining *Ovis* sequences obtained from both wild (*O. canadensis*, *O. dalli*) and domestic sheep (*O. aries*) diverged following the split. The tree places Ovar-DRA*0201 with the *Orda-DRA* (Scimitar Horned Oryx) and *Cota-DRA* (Blue Wildebeest), rather than in the group containing the other three *Ovar-DRA* alleles. The relationship between Ovar-DRB1*0201 and the Oryx and Wildebeest *DRA* sequences is supported by high posterior probability values.

#### Evidence of positive selection using modified Branch-Site models

This analysis identified a high probability of positive selection associated with polymorphic sites in the Ovar-DRα*0201 molecule. Ten sites where the probability of the ratio of nonsynonymous to synonymous substitutions exceeds one were found on the branch leading to Ovar-DRA*0201, seven of which locate to the alpha 1 domain (Figure 2 and Table 4). By comparison with those amino acids also associated with the peptide binding groove, G11, V72 and Y76 also appear to be under positive selection (Table 4). Other sites under selection M6, I12, also appear adjacent to residues predicted to form the antigen binding groove and these may also be implicated in structural changes associated with the repertoire of peptides presented to T cells. Sites under positive selection within the usually conserved alpha 2 domain; S152, C168, S169 may also be linked to structural changes associated with the formation of class II heterodimer or the binding of accessory molecules such as CD4.

**Table 4:** Amino acid sites under positive selection.

Site	Ovar-DRA*0201	Substitution	P value
6	M	V	0.963
11	G	E	0.969
12	I	F	0.989
14	V	L	0.983
72	V	I	0.970
74	T	I	0.979
76	Y	R	0.995
152	S	P	0.976
168	C	W	0.965
169	S	G	0.982

Numbering of amino acid sites under selection is as shown in Figure 2. P = the probability of the ratio of nonsynonymous to synonymous substitutions at each site being greater than one.

To check for positive selection on the other 18 branches in the tree, a Branch-Sites analysis was carried out. For 13 branches, the Likelihood ratio test (LRT) statistic was zero and the magnitude was between zero and 0.93 for 4 other branches. The only significant branch was the one connecting the pig DRA outgroup to the ingroup (LRT test statistic of 6.35; p = 0.012). The LRT statistic for the Ovar-DRA*0201 branch was 6.60 (p = 0.010).

### Functional analyses

**Figure 4:**
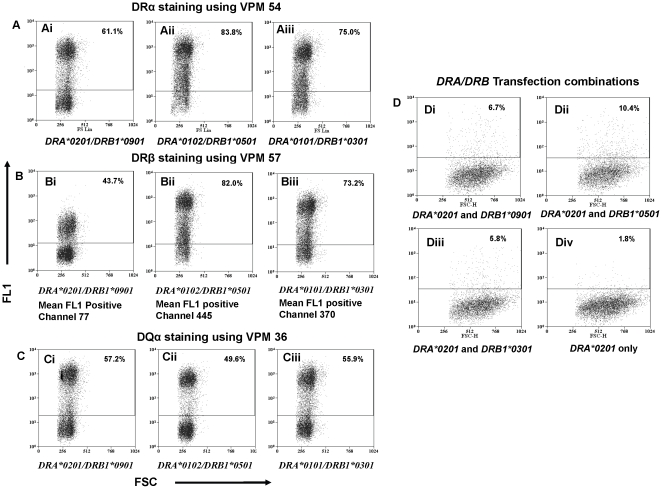
Surface expression of MHC class II DR. **4A** Peripheral blood mononuclear cells derived from sheep homozygous for i; DRA*0201/DRB1*0901, ii; DRA*0102/0501 and iii; DRA*0101/0301 using DRα chain specific monoclonal antibody VPM 54. Horizontal axis FSC, forward scatter (cell size), vertical axis FL1, fluorescence channel 1. The percentage of positive events above the cut-off set based of the unlabelled population is shown in the top right. **4B** Peripheral blood mononuclear cells derived from sheep homozygous for i; DRA*0201/DRB1*0901, ii; DRA*0102/0501 and iii; DRA*0101/0301 using DRβ chain specific monoclonal antibody VPM 57. **4C** Peripheral blood mononuclear cells derived from sheep homozygous for i; DRA*0201/DRB1*0901, ii; DRA*0102/0501 and iii; DRA*0101/0301 using DQα chain specific monoclonal antibody VPM 36. **4D** COS-7 cells transfected with haplotype matched and mismatched combinations of *DRA/B* genes. i; DRA*0201 and DRB1*0901, ii; DRA*0201 and *0501*, iii; DRA*0201 and *0301* and iv; DRA*0201 only.

Functional implications of Ovar-DRA*0201 diversity were investigated in animals homozygous for the DRA*0201-DRB1*0901 haplotype. Expression of DR protein on the surface of peripheral blood mononuclear cells was confirmed by flow cytometry using a DRα chain specific monoclonal antibody (Figure 4a). With only the DRA*0201 and DRB1*0901 genes present in this haplotype the DR protein must derive from these genes. The level and intensity of class II DR expression was compared in additional animals homozygous for the MHC haplotypes, DRA*0102-DRB1*0501 and DRA*0101-DRB1*0301. Comparable percentages of DR and DQ positive cells with similar staining intensities were identified in each (Figure 4a and 4c). Interestingly, labelling of the same cells with the DRβ chain specific antibody identified a reduction in the intensity of staining of DR positive cells in the DRA*0201 homozygous animal compared with DRA*01 animals (Figure 4b). This is likely to reflect a structural change in the DR molecule which alters the affinity of binding of the DRβ chain specific monoclonal antibody while the DRα chain specific mAb remains unaffected.

The failure to observe recombination between DRA*0201 and DRB1*0901 suggests that the integrity of this gene pair may be maintained by constraints limiting functional pairing of the products of these genes. We addressed this by transfecting different allelic combinations of *DRB1* into COS-7 cells along with DRA*0201. Individual α and β chains fail to get to the surface of transfected cells and expression at the cell surface can occur only if complementary α and β chains are present. As expected, DRB1*0901 complemented DRA*0201 and surface-expressed MHC class II was detected by flow cytometry (Figure 4d). However, DRA*0201 was also complemented by DRB1*0301, and DRB1*0501 indicating that DRA*0201 is not functionally restricted to only the DRB1*0901 allele. The reverse experiment where DRB1*0901 was co-transfected in turn with each of the *DRA* alleles also failed to identify any functional constraints linked to surface expression (data not shown).

## Discussion

Allelic diversity at MHC class II *DRA* loci in vertebrates is generally low with few non-synonymous substitutions. At the time of writing, the IMGT/HLA database (www.ebi.ac.uk/imgt/hla/index.html) holds 762 *HLA-DRB1* alleles. In contrast, the database holds only three *HLA-DRA* alleles, corresponding to two proteins that differ by only a single amino acid. Similarly, comparison of *B. taurus* [20] and *B. indicus* cattle *DRA* sequences revealed only three synonymous substitutions, despite evidence from mitochondrial sequence and microsatellite analysis that these subspecies diverged from a common ancestor between 200, 000 and 1 million years ago [18], [22], [23]. This suggests that the *DRA* locus is under considerable purifying selection. However, we have observed diversity at the *DRA* locus of domestic sheep that is far in excess of anything described in other vertebrate species. Our sequence and phylogenetic analyses, supported by haplotype and functional data, are all consistent with DRA*0201 representing an unusual allele at the *Ovar-DRA* locus rather than a novel class II MHC *DRA*-like locus. The coding region of Ovar-DRA*0201 differs from the reference *DRA* sequences by 20 nucleotides, of which 12 are non-synonymous. Six of these polymorphisms are unique when compared with orthologous sequences from a wide range of *Cetartiodactyl* species and two are predicted either directly (R76Y) or indirectly (V6M) to influence the structure of the peptide binding site and hence the range of peptides presented to CD4 +ve T cells. Furthermore, using MHC homozygous animals this *DRA* allele is shown to be linked to Ovar-DRB1*0901; an allele that also presents unusual patterns of sequence diversity in coding, regulatory and intronic regions [7]. These data are consistent with an independent evolutionary history for the Ovar-DRA*0201/Ovar-DRB1*0901 gene pair.

The evolutionary origin of this divergent gene pair is far from clear. The complex origin of domestic sheep is apparent from the presence of at least five distinct mitochondrial lineages [20], some of which cannot be traced to a wild ancestor [24], [25]. This diversity is likely to originate from geographically isolated subspecies of wild sheep that have hybridised as a result of human migrations over the 8–10 millennia since the initial domestication events in the Near East and Asia [26]–[28]. Frequent hybridization events are likely to have occurred between domesticated and local wild populations providing the high levels of MHC diversity evident in present day domestic populations as well as a degree of resistance to endemic disease and adaptation to local environmental conditions [29].

Cross-species hybridisation or ancient allelic lineages present in the ancestral founding population of sheep are the two most likely explanations for the origin of the unusual allelic pair. In the absence of the *DRA* data provided here, cross-species hybridisation was favoured by Schwaiger et al [16] to explain the divergence of the ESG2 group of *DRB1* alleles. Such an event could be responsible for the introgression of genetic material from an unidentified species into one of the founding populations of modern domestic sheep [16], [7] with balancing selection maintaining these unusual alleles in contemporary domestic sheep populations. However, with the inclusion of the *DRA* analysis the cross species hybridisation explanation now appears less likely. Analysis of *DRA* diversity in a wide range of *Cetartiodactyla* species failed to identify an extant species that could have supplied DRA*0201. Indeed, it identified a phylogenitic relationship between Ovar-DRA*0201 and Oryx/Wildebeest *DRA* sequences which is consistent with a much older lineage present prior to the speciation events that led to present day ruminants. Balancing selection appears not only to maintain high levels of MHC diversity at individual loci [30]–[32], but is likely to have played a role in the persistence over a long period of time of the unusual allelic diversity at the *DRA* and *DRB1* loci in sheep.

The relationship between the Oryx/Wildebeest *DRA* and Ovar-DRA*0201 supports the trans-species nature of MHC allelic lineages [31], [32]. However, some caution in interpreting data from conserved sequences is required as a limited number of substitutions may have a large effect on the predicted phylogenetic relationship. Two nucleotide substitutions corresponding to amino acids 72 and 74 of Ovar-DRA*0201 in addition to nucleotide substitutions at positions 46 and 117 have an important influence on the phylogenetic relationships. While these substitutions may each have arisen independently in sheep DRA*0201 and Oryx/Wildebeest *DRA* in our opinion this is however, more unlikely than shared ancestry. Indeed, Bayesian phylogenetic methodology provides a probability of 0.93 that these three *DRA* sequences are distinct from the other sequences in the dataset.

The DRB1*0901 allele is widely distributed in breeds including Finish Landrace [16], Merino [11], Polish Heath [33], Spanish Lataxa [34], Suffolk (AB017204, Aida Y. Unpublished) and Mongolian Argali, a representative of the *Ovis ammon species* [16]. While in these instances we are unable to confirm the presence of the DRA*0201 allele we predict that the presence of both is likely to be associated with some unknown selective advantage most likely linked with binding peptide antigen for presentation of to T cells.

MHC class II DR protein diversity within vertebrate populations is typically generated through an almost invariant DRα chain coupled with a broad range of highly polymorphic DRβ products. In this way, individuals that are heterozygous at the *DRB* locus generate two distinct DR class II molecules, each capable of presenting a distinct repertoire of pathogen-derived peptides to CD4^+^ T cells. Sheep heterozygous for both *DRA* and *DRB1* have the potential to double the number of functional DR molecules from two to four through cis and trans associations. Such an increase in the number of class II DR molecules may provide an advantage with respect to the ability to respond to pathogen infection. On the other hand, this might be balanced by a corresponding reduction in the repertoire of functional T cell receptors as a result of greater T cell depletion during thymic development [35].

In conclusion, unusual allelic diversity has been identified at the *DRA* locus in domestic sheep. Using MHC homozygous animals, we have demonstrated that DRA*0201 is paired with the equally divergent *DRB1* allele *0901, a representative of an evolutionary distinct family of *DRB1* alleles. The functional *A/B* gene pair shows no evidence of recombination but the individual *A* and *B* genes can complement expression of haplotype-mismatched *DRA* and *DRB1* alleles. Phylogenetic analysis of full-length transcripts and exon 2 fragments from a wide range of Cetartiodactyl species suggests an independent evolutionary history for this gene pair, which is likely to have been present in the ancestral founding population of sheep and has been maintained in present day sheep populations by balancing selection.

## Materials and Methods

### Animals and nucleic acid

All Scottish Blackface, Suffolk and Bluefaced Leicester sheep were derived from the flocks maintained at the Moredun Research Institute, (MRI) Edinburgh, UK. Peripheral blood mononuclear cells (PBMC) were prepared by density centrifugation according to standard methodologies. Poly-adenylated mRNA was extracted from 2×10^6^ PBMC using the Dynabeads mRNA direct kit (Dynal Oslo, Norway). First strand cDNA was prepared using the Promega reverse transcription system in a 40 µl reaction containing 5 µl of the 20 µl poly A RNA preparation. Genomic DNA from Suffolk/Texel cross and Texel/British milk cross sheep was provided by Dr Chris Cousins, (MRI). Genomic DNA from other representative species within the Caprinae subfamily; *Capra hircus Montecristo*, *Capra Falconeri Heptner*, *Ovis dalli*, *Ovis canadensis* and *Budorcas taxicolor* were provided by Massimo Palmarini, (University of Glasgow). Genomic DNA from Chamois, (*Rupicapra rupicapra*), Blue Wildebeest (*Connochaetes taurinus*), Scimitar horned Oryx (*Oryx dammah*) and Alpaca (*Lama pacos*) was prepared from diagnostic tissue samples supplied by Kim Willoughby (MRI). Genomic DNA was prepared from whole blood using the Qiagen DNAeasy kit according to the manufacturer's instructions. Genomic DNA from Sperm whale (*Physeter macrocephalus*), common (*Delphinus capensis*) and Rissos dolphin (*Grampus griseus*) was prepared from autopsy tissue obtained from animals stranded on the Scottish coast.

Kenyan Boran (African Zebu *Bos indicus*) and Sahiwal cattle (Asian *B. indicus*), which originate from the Punjab region along the India-Pakistan border, were maintained at the International Livestock Research Institute's Kapiti Ranch and the Kenyan Agricultural Research Institute's Naivasha field station, respectively. Peripheral blood was collected from Boran and Sahiwal cattle pre-selected for allelic diversity at the *BoLA-DRB3* locus by PCR-RFLP [13]. Poly-adenylated mRNA extraction and cDNA preparation was as described for sheep.

### Amplification of full-length ovine and bovine *DRA* transcripts

Full length ovine and bovine MHC class II *DRA* transcripts were amplified by PCR and cloned into the pGEM-T Easy vector (Promega). PCR reactions were carried out in a final volume of 50 µl containing 200 nM of each primer (Table 5), 1U Platinum *Taq* polymerase (Invitrogen Ltd, Paisley, UK) and 4µl of the reverse transcription reaction. The cycling profile consisted of 35 cycles of 1 min at 94°C, 20 s at 60°C and 2 min at 68°C, and one cycle of 1 min at 94°C, 20 s at 60°C and 10 min at 68°C. Amplified products were gel-purified and cloned into the vector according to the manufacturer's instructions. Multiple clones (from at least two different PCR reactions) were selected and sequenced in both directions to verify sequence.

**Table 5:** PCR Primers.

Primer Specificity	Forward primer	Reverse primer
*BoLA-DRA* *	caccaaagaagaaaatggcc	tgagacccacttgaagtttactgtatt
*Ovar-DRA*	cacctcaagacaccaaagaag	ctctctaacaaagtccgttacc
*Ovar-DRB1*	cacctctccctctctatcctctgctg	ggttcttccttgagtgtgacc

*From [14].

### Amplification of *Cetartiodactylia DRA* exon 2

Exon 2 of the *DRA* gene was amplified from genomic DNA from a wide range of *Cetartiodactylia* species using intronic primers [36]. PCR products were sequenced directly in both directions using the same primers.

### Generation of mammalian expression constructs

Ovine MHC class II *DRA* and *DRB* genes were amplified from pGEM-T Easy vector clones and subcloned into the eukaryotic expression vector pcDNA3.1/V5-His-TOPO (Invitrogen). PCR reactions were carried out as described for the original amplification using primers listed in Table 5. All clones were sequenced in both directions to verify integrity. Plasmid DNA for transfection was prepared from 50 ml bacterial cultures using the SNAP midi kit (Invitrogen), precipitated in ethanol and resuspended under aseptic conditions to a concentration of 500 ng/ul in sterile water.

***Sequence analysis*** *DRA* contigs were assembled using the SeqManII program of the DNASTAR package. The full length and exon 2 sequences have been deposited in the EMBL database and assigned the accession numbers FM986335–FM986352 detailed in Table 6.

**Table 6:** Origin of *DRA* full length and exon 2 sequences.

Accession number	Species Name	Common Name	Allele Name	Sequence
FM986335	*Ovis* aries	Domestic sheep	*Ovar*-DRA*0101	Full length transcript
FM986336	*Ovis* aries	Domestic sheep	Ovar-DRA*0102	Full length transcript
FM986337	*Ovis* aries	Domestic sheep	Ovar-DRA*0201	Full length transcript
FM986338	*Bos* indicus	Zebu cattle	BoLA-DRA*01013	Full length transcript
FM986339	*Bos* indicus	Zebu cattle	BoLA-DRA*01014	Full length transcript
FM986340	*Ovis* canadensis	Big Horn Sheep	Ovca-DRA*0101	Exon 2
FM986341	*Ovis dalli*	Dalli sheep	Ovda-DRA*0101	Exon 2
FM986342	*Oryx* dammah	Scimitar Horned Oryx	Orda-DRA*0101	Exon 2
FM986343	Connochaetes *taurinus*	Blue Wildebeest	Cota-DRA*0101,	Exon 2
FM986344	Rupicapra *rupicapra*	Chamois	Ruru-DRA*0101	Exon 2
FM986345	*Budorcas* taxicolor	Takin	Butu-DRA*0101	Exon 2
FM986346	*Capra* falconeri	Markhor	Cafa-DRA*0101	Exon 2
FM986347	Cervus *elaphus*	Red deer	Ceel-DRA*0101	Exon 2
FM986348	*Rangifer* tarandus	Reindeer	Rata-DRA*0101	Exon 2
FM986349	Lama *pacos*	Alpaca	Vipa-DRA*0101	Exon 2
FM986350	*Delphinus* capensis	Common Dolphin	Deca-DRA*0101	Exon 2
FM986351	Grampus *griseus*	Risso's dolphin	*Grgr*-DRA*0101	Exon 2
FM986352	*Physeter* catodon	Sperm Whale	*Phca*-DRA*0101	Exon 2

Database accession numbers and species of origin of the full length and exon 2 *DRA* sequences generated though this study.

DNA Multiple alignments were produced using the CLUSTALW program using the profile alignment option to merge alignments of the long (fill length) and short (exon 2 only) sequences. Alignments of the corresponding protein sequences were used to check the DNA alignments. The use of a combined alignment of long and short sequences improves the phylogenetic tree estimate if the alignment is of high precision (i.e. long gaps in the correct position). Such a combined alignment yields an improved tree estimate as all available data is used to resolve the species relationships. Modern statistical methods, namely Bayesian inference and Maximum Likelihood, handle missing values efficiently. On the contrary, distance-based methods such as Neighbour–Joining are expected to perform poorly as pairwise distances do not allow information on gaps to be used optimally.

### Phylogenetic inference

#### Selection detection using the Branch-Site Test

To detect positive Darwinian selection in the protein-coding multiple alignment we used the statistical methods available in the PAML package [37] developed by Ziheng Yang. PAML supplies a range of modern statistical tests to detect positive selection based on the ratio (omega) of nonsynonymous to synonymous substitutions. When no selection has acted, omega is expected to be one. The most common type of selection, negative selection will result in omega being less than one. PAML uses a likelihood ratio test to produce a probability that omega is greater than one.

We used the recently developed Branch-Site models [38] plus an associated likelihood ratio test (LRT) [39]. This approach has been applied to data similar to *DRA* namely the *DRB* gene by the developers of the method [40]. With the Branch-Site approach, the branches to be assessed for the influence of positive selection, i.e. the “foreground” branches, require to be chosen in advance. We then compared, by use of the LRT, a branch–site model allowing positive selection on the foreground branches with a simpler model that does not. When there is a lack of a clear biological hypothesis to guide the choice of the foreground branch, one approach is to carry out many analyses by selecting each branch in turn as the foreground branch. In this case, we are primarily interested in the branch leading to Ovar-DRA*0201. However, it is prudent to test the other branches, especially those leading to the other sheep *DRA* sequences. Positive selection analysis was carried out on the full alignment length which provides the largest number of sites.

#### Estimating phylogenetic trees

The full length cDNA alignment and exon 2 DNA alignments were merged using the profile alignment facility within the Clustalw program to create a 34 by 759bp alignment. Prior to phylogenetic tree estimation, the codon position model was optimised using the model selection feature in the TOPALi v2 package [41] which produces improved estimates of Likelihood values and derived statistical quantities (AIC, BIC). A nucleotide substitution model was estimated for each of the three codon positions: HKY+G (position 1), HKY+I (position 2), and HKY+G (position 3). These models were then used to estimate a Bayesian phylogenetic tree using the MrBayes program [42] launched from TOPALi v2.5. The MrBayes settings were 2 runs of 625,000 generations and a burn-in period of 125,000 generations, with trees were sampled every 100 generations. Convergence was assessed using the PSRF statistics produced by MrBayes for each parameter value, with a value of 1.00 denoting complete convergence. The maximum PSRF value encountered was 1.07, with the vast majority of values (123 out of 135) less than 1.01.

#### Predictive analysis of amino acid polymorphism

The Sorting Intolerant from Tolerant (SIFT) BLink program, (http://sift.jcvi.org), [43], analyse alignments of orthologous, or paralogous sequences and predicts whether an amino acid substitution will affect protein function. The Ovar-DRA*0101 protein sequence was used as the input sequence with each of the substitutions associated with the DRA*0201 sequence targeted. The alignment of sequences generated for the analysis was edited so as not to include the Ovar-DRA*0201 sequence and to only include orthologous and paralogous full-length sequences. A scaled probability matrix is generated for the target protein with a threshold of 0.05 used to define intolerant from tolerant amino acid substitutions.

### Nomenclature

Domestic sheep (*Ovis aries*) *DRA* alleles are designated in accordance with the MHC nomenclature system proposed for all vertebrates [44]. This system is used for all species and allele designations described herein, with a few historical exceptions such as HLA for the human MHC and BoLA for the cattle MHC. To maintain consistency with other *Ovar-MHC* loci (http://www.ebi.ac.uk/ipd/mhc), the following nomenclature for alleles at the *Ovar-DRA* locus was adopted. The first two digits following the species and locus designation (*Ovar-DRA*) represents the allelic family (Ovar-DRA*01, *02 etc). Alleles within a family differ by no more than four amino acids over the entire coding region. The next two digits indicate coding change within the allelic family (Ovar-DRA*0101) and a fifth (Ovar-DRA *01011) may be used to indicate silent or synonymous substitutions. The reference sequence Ovar-DRA*0101 was obtained from a genomic clone [15] and validated from a number of full length transcripts described herein.

### Transfection and detection of MHC gene expression

Ovine MHC class II *DRA* and *DRB* genes were co-transfected into COS-7 cells using the DEAE-dextran method [45]. Products of transfected class II MHC genes were detected at the cell surface after 72 hours by indirect immunofluorescence using a combination of monoclonal antibodies (mAb) VPM 54 and VPM 57, which recognise monomorphic determinants on the ovine MHC class II DRα and DRβ chains respectively [46]. Alexa fluor 488-conjugated goat anti mouse IgG (Molecular Probes, 1∶1000 final dilution) was used as the secondary reagent. Sample data were acquired using a FACSCalibur flow cytometer equipped with a 488 nm argon-ion laser and analyzed using CellQuest (Becton Dickinson) software. Expression of class II MHC on the surface of PBMC was determined by indirect immunofluorescence using the DRα chain specific mAb VPM 54, DRβ chain specific mAb VPM 57 and the DQα chain specific mAb VPM 36 [46].

## Supporting Information

Figure S1 Nucleotide sequence of full length *Ovar-DRA* transcripts. Nucleotide sequence of full length *Ovar-DRA* transcripts aligned with orthologous sequences from domestic goat (*Capra hircus*, [48], cattle (BoLA-DRA*01011, [14], DRA*01013 and DRA*01014 this paper), water buffalo (*Bubalus bubalus* DQ016629, unpublished) and domestic Pig [49]. Nucleotide identity is represented by dots (.) and the second exon is shaded.
(1.57 MB TIF)

Figure S2 DRA exon 2 sequences within the order cetaryiodactylia: Nucleotide sequences of DRA exon 2 derived from 33 species within the order cetaryiodactylia. Species designations associated with MHC nomenclature are as follows; Bibo, Bison bonasus (European Bison); Boga, Bos gaurus (Gaur); Boja, Bos javanicus (Banteng); Bubu, Bubalus bubalis (Asian Water Buffalo); Bude, Bubalus depressicornis (Lowland Anoa); Buta, Budorcas taxicolor (Takin); Cahi, Capra hircus, (Domestic Goat); Cafa, Capra falconeri (Markhor); Ceel, Cervus elaphus (Red Deer); Cota, Connochaetes taurinus (Blue Wildebeest); Deca, Delphinus capensis (Common Dolphin); Grgr, Grampus griseus (Risso's Dolphin); Orda, Oryx dammah (Scimitar Horned Oryx); Ovar, Ovis aries (Domestic Sheep); Ovca, Ovis canadensis, (Canadian Bighorn Sheep); Ovda, Ovis dalli (Dalli Sheep); Ovmo, Ovibos moschatus (Musk Ox); Phca, Physeter catodon (Sperm Whale); Ruru, Rupicapra rupicapra (Chamois); Rata, Rangifer tarandus (Reindeer); SLA, Swine Leucocyte Antigen (Pig); Syca, Synserus caffer (African Buffalo); Vipa, Vicugna pacos (Alpaca).
(1.57 MB TIF)
